# Upholding or Breaking the Law of Superposition in Pharmacokinetics

**DOI:** 10.3390/biomedicines12081843

**Published:** 2024-08-13

**Authors:** Malaz Yousef, Jaime A. Yáñez, Raimar Löbenberg, Neal M. Davies

**Affiliations:** 1 Faculty of Pharmacy and Pharmaceutical Sciences, University of Alberta, Edmonton, AB T6G 2E1, Canada; malaz@ualberta.ca (M.Y.); raimar@ualberta.ca (R.L.); 2 Escuela de Posgrado, Universidad Internacional Iberoamericana, Campeche 24560, Mexico; jaime.yanez@unini.edu.mx

**Keywords:** pharmacokinetics, superposition, steady state, linearity, multiple dosing

## Abstract

The law of superposition underpins first-order linear pharmacokinetic relationships. Most drugs, therefore, after a single dose can be described by first-order or linear processes, which can be superposed to understand multiple-dose regimen behavior. However, there are a number of situations where drugs could display behaviors after multiple dosing that leads to capacity-limited or saturation non-linear kinetics and the law of superposition is overruled. This review presents a practical guide to understand the equations and calculations for single and multiple-dosing regimens after intravenous and oral administration. It also provides the pharmaceutical basis for saturation in ADME processes and the consequent changes in the area under the concentration–time curve, which represents drug exposure that can lead to the modulation of efficacy and/or toxic effects. The pharmacokineticist must implicitly understand the principles of superposition, which are a central tenet of drug behavior and disposition during drug development.

## 1. Introduction

Although there are some drugs that can be taken acutely as a single dose, such as anti-migraine drugs [1,2,3,4] or hypnotics [5,6,7,8] for a limited period of time, the vast majority of drugs, such as antibiotics [9,10,11,12], anti-diabetics [13,14,15], and blood pressure medication [16,17,18], are designed to be repeatedly administered orally. Chronic disease states often require a multiple-dose regimen to enable the accumulation of the drug and attain steady-state concentrations [19,20,21]. The law of superposition or sometimes referred to as the principle of superposition enables the understanding of the pharmacokinetics of a drug after a single dose and for the accurate prediction of the steady-state conditions to be made for repeated dosing or, potentially, vice versa [22,23]. While some scientists, particularly in the pharmaceutical industry, may have training during pharmacy education on pharmacokinetics, others have a varied biomedical background and are less versed in the underlying pharmacokinetic theorems. In 21st century drug development, there are a myriad of pharmacokinetic technology programs and simulation software that will enable investigators to take and input single-dose pharmacokinetic concentration–time data and predict steady-state concentrations after a specific dose regimen. Modeling programs offer the advantages of large data handling capacities and execution speed; however, the conceptual and mathematical limitations to this modeling and the interpretation of the results must be clearly understood and delineated.

The law of superposition is a fundamental aspect of pharmacokinetics, and it is often dealt with in a cursory manner in textbooks. Few articles have tried to deal with a conceptual understanding of superposition in a practical pharmacokinetic manner to demonstrate the application of the principle [19,22,23,24,25,26,27,28,29,30,31,32]. In this article, we first present a review of the principles of superposition and then discuss its application to multiple dosing. Finally, we apply this principle through simulations to gain an understanding of where the law of superposition does not hold and illustrate the non-linearity in the processes that can, in fact, overturn this law.

### Law of Superposition

Pharmacokinetically, the body can be considered to be a construct function that in mathematical terms converts the input (dose rate) into an output or response (plasma concentration of drug), as a function of time [32,33,34]. A linear system is characterized by input–output relationships that are completely defined by one or more linear equations, exhibiting properties of homogeneity and additivity. Linear systems applied to the field of pharmacokinetics were explored by Cutler [24,25], where the differential equations involved define a function. The most important feature of a linear system is that it is additive and obeys the law of superposition, where the output of a linear system equals the sum of the outputs of separately applied inputs [23]. The application of a linear model is contingent upon the condition that the concentrations involved are within a range where saturation effects are absent, indicating a limited scope of inputs [34,35]. For a specific input range, the linearity of the system can be experimentally tested to either reject or disprove the null hypothesis.

**Figure 1:**
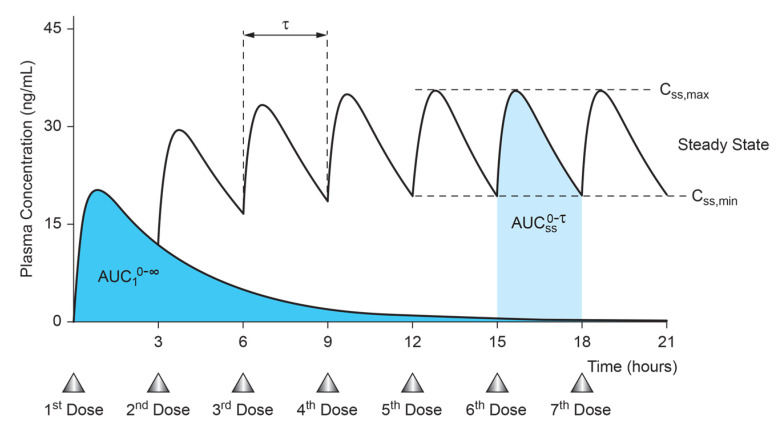
Concentration time profile after a single dose and multiple dosing when reaching the steady state.

Simply put, the law of superposition is a mathematical concept that enables pharmaceutical scientists to predict concentration–time data [36,37]. It has, however, an underlying assumption that the preceding dose of a drug and its pharmacokinetics do not alter or affect in any way subsequent doses of a drug and the expressed pharmacokinetic profile [38,39,40]. In practice, the plasma concentration–time data after the first, second, or n^th^ dose will be superimposed by the plasma concentration reached after the (n − 1)^th^ dose [39]. For practical purposes, the area under the plasma concentration–time curve after a single dose is equal to the area under the plasma concentration–time curve after multiple doses when steady-state concentrations are attained [39] (Figure 1).

The law of superposition allows the pharmacokineticist to project the plasma drug concentration–time curve of a drug after multiple consecutive doses, based on the plasma drug concentration–time curve obtained after a single dose [39,40]. It is important in a clinical setting to have the patient exposed to the target drug concentration without excessive fluctuation and drug accumulation outside of the therapeutic window [37]. Following a single dose, the extent of overall drug exposure over time can be quantified by estimating the area under the concentration–time curve from zero to infinity (AUC_0−∞_). During multiple dosing, the area under the concentration–time curve over a steady-state dosing interval (AUC_0−τ_) serves as a measure of overall drug exposure. When the clearance (CL), the aggregate of all the elimination processes, remains constant (i.e., it does not change with increasing drug concentrations), the AUC_0−τ_ at the steady state will be equivalent to the AUC_0−∞_ following a single-dose administration [39,41].

In the analysis of AUC data in pharmacokinetics, the law of superposition provides a simple test for detecting the presence of non-linear processes that do obey the law, i.e., processes that are first-order processes [34]. Practically, a greater than or less than response, in terms of the area under the drug concentration–time curve (AUC) with repeated doses is the expressed result of the involvement of zero-order processes [39]. AUC equivalence presumes that the dose administered as a single dose matches the dose given in a multiple dose. This is useful for determining pharmacokinetic parameters [23,27,42]. For example, after a single IV bolus dose, the total body clearance (CL) can be computed using Equation (1).

(1) $$CL=\frac{Dose}{{AUC}_{0-\infty }}$$

AUC equivalence enables the estimation of clearance under steady-state conditions using the same equation, but using AUC_0−τ_ instead of AUC_0−∞_. The latter clearance estimate is often referred to as the steady-state clearance (CL_ss_). In the case of a single oral dose, the AUC is calculated taking into consideration the bioavailability (F) of the drug when using CL_total_ (Equation (2)) or taking into consideration the fraction unbound in blood (f_u_) when using the intrinsic clearance (CL_int_) [22] (Equation (3)).

(2) $$AUC=\frac{F\times Dose}{{CL}_{total}}$$

(3) $$AUC=\frac{F\times Dose}{fu\times {CL}_{int}}$$

**Figure 2:**
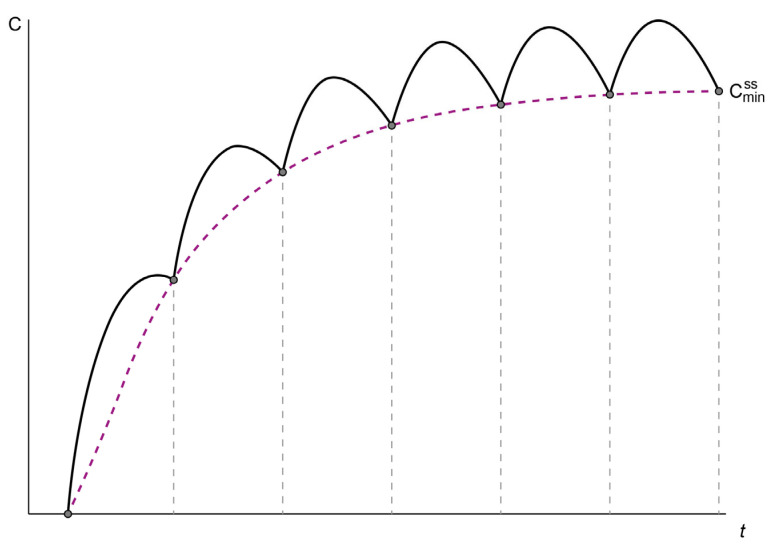
Concentration–time profile of an ideal scenario after multiple-dose regimen 132, administered at the same time interval and reaching a steady state. Modified from Wang et al. [19].

It becomes readily apparent in Figure 2 that in multiple dosing the concentrations oscillate at steady state between a minimum concentration at a steady state (C_SSmin_) and a maximum concentration at a steady state (C_SSmax_) and that an average steady-state concentration C_av,SS_ can be calculated [19].

The drug clearance can be determined by dividing the dose by either of the aforementioned two AUC values, or by dividing the dosing rate by the average steady-state concentration (C_av,ss)._ The C_av,ss_ is calculated by dividing either of the two AUC values by the dosing interval (Tau, T) [27]. Thus, the average steady-state plasma level (C_p,ss_) can by calculated by Equation (4).

(4) $$C_{p,ss}=\frac{{AUC}_{0-\infty }}{T}$$

Superposition is based on mathematical equations describing linear processes [34,35]. Both experimental data and drug development results can uphold or refute the mathematical relationship [43]. Pharmaceutical development that relies solely on equations or theoretical superposition can be misleading or incorrect. There are observational limits to when superposition occurs and when it may deviate from linearity. Ab initio superposition involves multiple untested assumptions that might not hold for a particular drug in a particular disease state, as discussed in further detail below. Modeling approaches required for predictive analytics, including superposition, can utilize simple inductive reasoning and are only sometimes correlative with observational findings [30]. Further, a causality-based understanding of drugs is achieved through deductive reasoning and the application of a scientific method.

The law of superposition would predict that for a drug, the AUC after a single dose is equal to the AUC at the steady state. Therefore, Equation (5) would hold true.

(5) $$\frac{{AUC}_{SingleDose}}{{AUC}_{SS}}=1$$

This is independent of a compartmental model and is simply a function of linearity. Furthermore, the CL (after IV administration) or CL/F (after oral administration) following a single dose, will equal the clearance values at the steady state. In the case of oral administration, the bioavailability remains constant between a single dose and the steady state. The AUC at the steady state (AUC_SS_) can be defined as in Equation (6).

(6) $${AUC}_{SS}=\underset{n\to \infty }{\lim}\int_{t_{n}}^{t_{n+1}}C_{n}\left(t\right)dt$$

Non-linearity can cause the AUC_SS_ to differ from the AUC_Single Dose_ [32,43], this will be discussed later in the section on breaking the law of superposition. Most drugs will exhibit some deviation from the ideal accumulation, as described [37]. Nevertheless, comprehending the principle of superposition permits reasonable predictions of the pharmacokinetic behavior of drugs under repeat-dose conditions for a broad range of compounds. This understanding proves especially beneficial when transitioning from single-dose studies to repeat-dose studies during the initial stages of drug development. Additionally, this knowledge facilitates the design of repeat-dose regimens that achieve the desired drug concentrations efficiently and reliably within a clinically acceptable timeframe. When determining multiple-dose regimens, it is crucial to assess whether successive doses will impact the effects of previous doses. The principle of superposition assumes that earlier doses do not influence the pharmacokinetics of later doses. As a result, the blood levels following the second, third, or n^th^ dose will overlay those achieved after the (n − 1)^th^ dose [39]. In addition, the AUC_0−∞_ following single-dose administration is equal to the steady-state area between doses (Figure 1). The disposition processes after an oral dose are absorption, distribution, metabolism, and excretion (ADME). Therefore, the superposition principle is applicable when the ADME processes exhibit linear or first-order kinetics. With this approach, the concentrations after multiple doses can be determined by summing up the concentrations from each dose [24,25,42]. Furthermore, doubling the dose will result in proportional doubling of the concentrations for each time. However, this principle does not apply when any of the disposition processes are non-linear [24,25,34].

**Figure 3:**
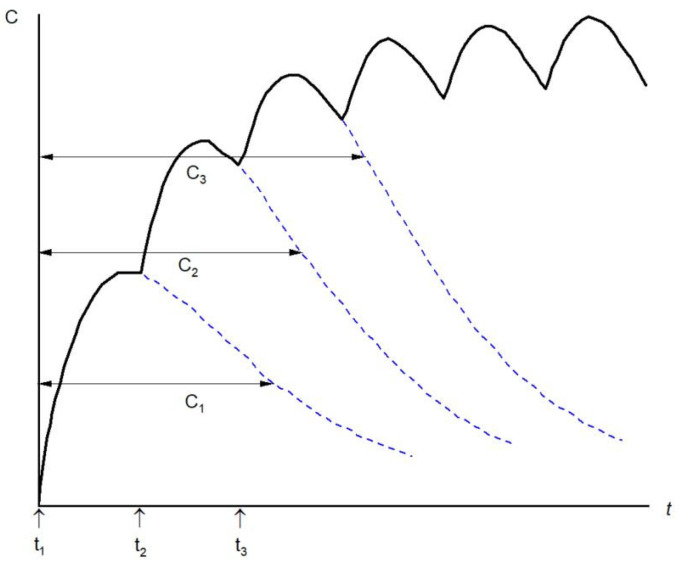
Representation of a concentration–time profile after six sequential doses administered at the same time interval until the steady state. Modified from Wang et al. [19].

Mathematically a single-dose concentration–time curve C(t) is denoted as a function over time f(t) [42]. Multiple dosing occurs when the administered doses remain constant over time, where the drug is administered at fixed time intervals (tau) (Figure 3). The concentration at time t after n doses is equal to the sum of the concentration at t after n − 1 doses and the single-dose concentration at t − t_n_ (from the last dose) [22]. Therefore, the contribution of the last dose can be superimposed to that of the first n − 1 doses [22]. The concentration–time curve C_n_(t) is described in Equations (7) and (8).

(7) $$C_{n}\left(t\right)=C_{n-1}\left(t\right)+f(t-t_{n})$$

(8) $$C_{n}\left(t\right)=\sum_{i=1}^{n}f(t-t_{i})$$

## 2. Modeling a Hypothetical Drug “Canadamycin”

### 2.1. Pharmacokinetics after a Single Intravenous Dose

For practical purposes, we named our hypothetical drug Canadamycin, which was to be administered via the intravenous (IV) route at a dose of 100 mg. It has a volume of distribution of 5 L, a half-life of 4 h, and dosing interval (tau) of 8 h. Therefore, a typical three times daily dosing (TID) is required. The C_0_ was estimated to be 20 mg/L (µg/mL), after dividing the dose by the volume of distribution (100 mg/5 L).

A direct intravenous bolus injection into a vein results in the near instantaneous achievement of C_0_, followed by a first-order reduction in the concentration. This route is frequently employed for drugs administered in hospital clinics or emergency settings, where a quick onset of action is critical. Pharmacokinetics textbooks typically utilize this approach to elucidate key concepts associated with drug accumulation [37,39]. Regardless of the administration route, as the number of doses progressively increases during a therapeutic drug regimen, drug concentrations within the dosing intervals (tau) will eventually stabilize, forming a plateau [42]. This phenomenon consistently occurs under the law of superposition, when conditions exhibit constant total clearance. At this plateau, the rate of drug administration matches the rate of drug elimination. When this equilibrium is achieved, plasma concentrations are considered to be at a steady state [42].

After a single IV bolus dose administration, the concentration of the drug will begin to decline and will be removed (cleared) and eliminated by the body. The concentration will decrease over time in terms of the half-life (the time required for the concentration to decline by 50%). Canadamycin has a half-life of 4 h, meaning that 8 h post-dose we would observe a concentration of 5 mg/L, a concentration of 1.25 mg/L at 16 h post-dose, and a concentration of 0.3124 mg/L at 24 h post-dose, etc. (Table 1). If no further doses are administered, the concentration will continue to decline by an additional 50% every 4 h. In this scenario, each subsequent dose is given before the previous dose is fully cleared from the body. As a result, the concentration at the beginning of the next dose is the sum of the drug amount remaining in the body (C_min_, denoted as Cτ above), plus the C_max_ from a single IV bolus dose (or C_0_). This cumulative effect of drug concentrations from previous doses and additional doses is the principle of superposition, which implies that each dose will additively influence the overall concentration.

Drug accumulation has a limit because, as the plasma concentration increases, the amount of drug eliminated (1 − r) during the dosing interval also grows. This is due to the fact that the elimination rate is directly proportional to the drug amount in the body, multiplied by the rate constant for first-order elimination [21,42]. As seen above, accumulation will occur if the dosing interval is less than 6 half-lives. Accumulation is inversely proportional to the fraction of the dose lost during an interval. The accumulation factor is equal to 1 over the fraction of the dose lost during a dosing interval = 1/(1 − fraction remaining) [21,42]. If a drug is given once every half-life, in the case of Canadamycin every 4 h, the accumulation of the dose is 2 [1/(1 − 0.5)]. If Canadamycin is administered every 8 h (or 2 half-lives), the accumulation factor is 1.33 [1/(1 − 0.25)], since there would be 25% of the drug remaining.

When a drug is administered at a fixed dose and a regular interval, as in multiple-dose regimens, the amount of drug in the body will increase and then plateau at a mean plasma level higher than the peak concentration (C_0_). If the second dose is administered before the previous dose is entirely eliminated, drug accumulation will occur. This accumulation is quantified by the R index and is influenced by the elimination constant and the dosing interval, but it is independent of the dose itself [21,42]. For a drug administered in repeated oral doses, the time required to reach the steady state depends on the elimination half-life of the drug. This time is independent of the size of the dose, the length of the dosing interval, and the number of doses [39]. For instance, if the dose or dosing interval of a drug is modified, the time needed to reach the steady state remains unchanged, but the final steady-state plasma level adjusts proportionately. Consequently, the blood concentration after the second, third, or n^th^ dose will superimpose the blood concentrations attained after the (n − 1)^th^ dose.

The principle of superposition allows for the prediction of the plasma drug concentration–time profile after multiple consecutive doses, by utilizing the plasma concentration–time data from a single dose. This method is based on two key assumptions: that the drug follows first-order elimination kinetics, and that its pharmacokinetics remain unchanged with repeated dosing. As a result, the plasma concentration after multiple doses can be forecasted from those observed after an initial dose. The first-order pharmacokinetics can apply to multiple dosing; consider Equation (9) to describe the plasma concentration at any time after a single IV bolus drug administration dose that follows linear pharmacokinetics.

(9) $$C\left(t\right)=\sum_{1}^{n}A_{i}e^{-t/\tau j}=\sum_{1}^{m}A_{i}e^{-ajt}$$

where A_i_ is the concentration coefficient, τ_j_ is the time constant, and a_j_ is the rate constant (a_j_ = 1/τ_j_).

The concentration at the end of the first dosing interval (tau) is described in Equation (10). Equation (11) describes the concentration at the start of the second interval, while Equation (12) describes the concentration at the end of the second dose interval.

(10) $$C_{p1}^{\tau }=C_{p1}^{0}e^{-k_{el}t}$$

(11) $$C_{p2}^{0}=C_{p1}^{\tau }{+C_{p1}^{0}=C_{p1}^{0}e}^{-k_{el}t}+C_{p1}^{0}$$

(12) $$C_{p2}^{\tau }=\left[{C_{p1}^{0}e}^{-k_{el}t}+C_{p1}^{0}\right]e^{-k_{el}t}$$

As described in Table 1, there is a fraction of the initial plasma concentration remaining at the end of the dosing interval. Intuitively there is both a fraction lost (R) and a fraction remaining in the body (1 − R) for every dosing interval. The remaining fraction in the body before the next dose depends only on the first-order rate constant of elimination and the time after the dose, as described in Equation (13).

(13) $$R=e^{-k_{el}t}$$

Thus, replacing R in Equation (12) to describe the concentration at the end of the second dose interval is shown in Equation (14). Equation (15) describes the concentration at the start of the third dose interval.

(14) $$C_{p2}^{\tau }=C_{p1}^{0}R+C_{p1}^{0}R^{2}$$

(15) $$C_{p2}^{0}=C_{p1}^{0}+C_{p1}^{0}R+C_{p1}^{0}R^{2}$$

**Figure 4:**
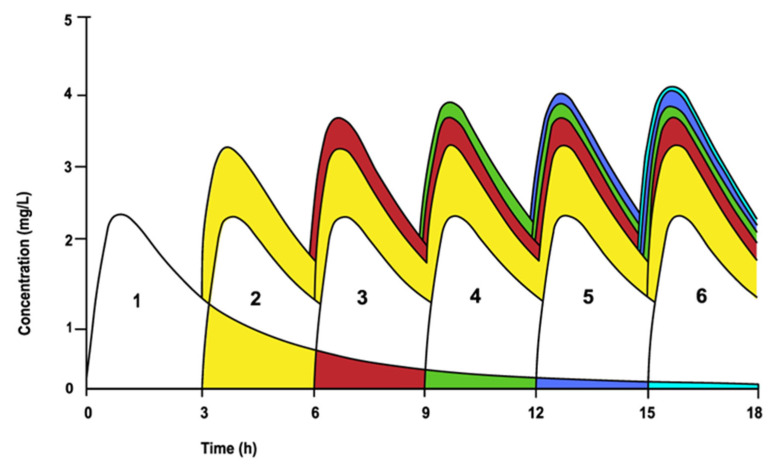
The law of superposition used to generate the concentration profile of multiple dosing from the profile of a single dose. The area of the first dose during the interval of the second dose adds to the area of the second dose, and so on. Modified from Van Rossum and de Bie [34].

These fractions of the drug that remain in the body before the next dose mathematically form a geometric series of remaining concentrations, with each term R times the preceding term. This leads to drug accumulation. In the case of multiple dosing, it appears that the concentration profiles of the individual doses can be totaled or summed up over time (Figure 4). The steady state can then be calculated by the addition of areas, without the need to use any pharmacokinetic model, provided that first-order linearity holds [34].

### 2.2. Pharmacokinetics after Multiple Intravenous Dosing

In linear pharmacokinetic systems, the drug concentration achieved by a second dose is comparable to that produced by the first dose. For instance, when a second 100 mg IV dose of Canadamycin is administered at the 8-h mark, 5 mg/L of the drug remains from the first dose, as shown in Table 1. Consequently, the plasma concentration (C_p_) immediately after the second dose would be 25 mg/L, consisting of the 5 mg/L remaining from the initial dose and an additional 20 mg/L from the new dose. This results in a combined concentration of 25 mg/L. In linear systems, each subsequent 100 mg dose will generate a concentration similar to that of the first dose. However, the resulting concentration will be the sum of the residual concentrations from all prior doses and the concentration from the most recent dose. For example, administering a third dose at the 16 h mark will also produce an initial concentration of 20 mg/L. The concentration remaining from the second dose would be 5 mg/L, and the concentration remaining from the first dose would be 1.25 mg/L. When these concentrations are combined, the observed total concentration will be approximately 26.25 mg/L, calculated as 20 mg/L (from the third dose) + 5 mg/L (remaining from the second dose) + 1.25 mg/L (remaining from the first dose). This additive process of combining concentrations from multiple doses to determine the observed concentration exemplifies the principle of superposition [21,42]. The described pattern of superposition assumes that each dose behaves similarly, despite an increasing concentration. While simple additive superposition is generally applicable to most drugs, it is important to note that the superposition of exposures can become more complex if the pharmacokinetic behavior of each dose changes with an increasing concentration. This complexity arises in drugs with saturable clearance, such as phenytoin, where the pharmacokinetics do not remain consistent as the concentration increases [44]. For the remainder of this review, the simple additive superposition scenario will be used.

Consecutive multiple doses of a drug will lead to an increasing concentration in the body until a plateau, known as the steady state, is reached. At a steady state, the amount of drug administered with each dose is balanced by an equivalent amount of drug eliminated from the body between doses, resulting in a constant rate of drug input and output (rate in = rate out) [39]. For the majority of drugs, reaching a steady state typically takes about five half-lives. The time required to attain a steady state in a repeat-dose regimen is governed exclusively by the half-life of the drug and the dosing interval, as accumulation and the remaining fraction are influenced by specific pharmacokinetic equations. Although changing the dose or dosing interval can influence the concentration at the steady state, it does not affect the time needed to reach the steady state. Some drugs have prolonged half-lives, ranging from days to weeks or even longer. In such cases, waiting for five half-lives to achieve the desired steady-state concentration may not be necessary. When time is critical, as in the administration of antibiotics for critically ill patients, a loading dose can be used to reach the steady state more quickly [39].

For our hypothetical drug, Canadamycin, the C_max_ and C_min_ values using Equations (16) and (17), respectively, can be estimated taking into consideration the following calculations:

(16) $$C_{max}=\frac{C_{p1}^{0}}{\left(1-R\right)}=\frac{20}{\left(1-0.257\right)}=26.92\;mg/L$$

(17) $$C_{min}=\frac{C_{p1R}^{0}}{\left(1-R\right)}=C_{\max}R=26.92\times 0.257=6.92\;mg/L$$

Dose = 100 mg

V_d_ = 5 L

t½ = 4 h

τ = 8 h

C_0_ = 20 mg/L

kel = 0.693/t½ = 0.693/4 h = 0.17 h^−1^

$R=e^{-k_{el}t}$ = $e^{-0.17\times 8}$ = 0.257

Therefore, the plasma concentration will fluctuate between 26.92 mg/L and 6.92 mg/L during each dosing interval when the steady state is reached.

### 2.3. Pharmacokinetics after Multiple Oral Dosing

For oral dosing, the equations are more complex, but it is possible to model and predict the concentration with repeated doses and to estimate the plasma concentration and dosing regimens using superposition. The C_max_ value corresponds to the concentration at the peak time (tpeak) after reaching the steady state. In this case, C_min_ is estimated based on the assumption that the plasma concentration has peaked and e^−ka t^ is close to zero and that the next dose is administered once the absorption phase is complete, which is described in Equation (18) and a simplified version is represented by Equation (19).

(18) $$C_{min}=\frac{F\times Dose\times k_{a}}{V\times (k_{a}-k_{el})}\left[\frac{e^{-k_{el}\tau }}{1-e^{-k_{el}\tau }}\right]$$

(19) $$C_{min}=A\left[\frac{R}{1-R}\right]$$

The relationship between the loading dose and maintenance dose and, thus, drug accumulation during multiple-dose administration can be examined by analyzing the ratio between the minimum concentration at the steady state and the concentration during one dosing interval (τ) after the first dose, as demonstrated in Equation (20).

(20) $$\frac{C_{min}}{C_{p1}^{\tau }}=\frac{1}{1-e^{-k_{el}\tau }}=\frac{1}{\left(1-R\right)}$$

Equations have been developed that facilitate the calculation of the plasma concentration achieved following multiple oral administrations. To start, the plasma concentration achieved following a single oral dose that does not have flip-flop kinetics [45] can be described by Equation (21).

(21) $$C_{p}=\frac{F\times Dose\times k_{a}}{V\times (k_{a}-k_{el})}\left[e^{-k_{el}\tau }-e^{-k_{a}\tau }\right]$$

An intriguing outcome of this equation is that the average plasma concentration remains the same whether the dose is given as a single dose every dosing interval (τ) or divided into shorter dosing intervals.

For multiple oral dosing, we will use our hypothetical drug, Canadamycin, with an oral dose of 300 mg every 12 h, a bioavailability (F) of 100%, a volume of distribution of 30 L, and a half-life of 6 h.

Dose = 300 mg

V_d_ = 30 L

t½ = 6 h

τ = 12 h

kel = 0.693/t½ = 0.693/6 h = 0.116 h^−1^

We will estimate the required dose (maintenance dose) to be administered every 12 h to achieve an average plasma concentration of 15 mg/L using Equations (22) and (23).

(22) $$C_{p}=\frac{F\times Dose}{V\times k_{el}\times \tau }$$

(23) $$Dose=\frac{C_{p}\times V\times k_{el}\times \tau }{F}=\frac{15\times 30\times 0.116\times 12}{1}=624\;mg$$

The loading dose can be calculated using Equation (24), with the use of the R value.

$R=e^{-k_{el}t}$ = $e^{-0.116\times 12}$ = 0.25

(24) $$Loading\;Dose=\frac{Maintenance\;Dose}{1-R}=\frac{624}{1-0.25}=832\;mg$$

To estimate the fluctuations in the plasma concentration, the C_min_ can be calculated using Equation (25).

(25) $$C_{min}=\frac{F\times Dose}{V}\left[\frac{e^{-k_{el}\tau }}{1-e^{-k_{el}\tau }}\right]=\frac{1\times 624}{30}\left[\frac{0.25}{1-0.25}\right]=6.93\;mg/mL$$

Therefore, the plasma concentration would likely fluctuate between approximately 7 and 23 mg/L, with an average concentration of around 15 mg/L. In this case, the average concentration can be estimated using an approximation of C_max_ = [C_av_ + (C_av_ − C_min_)] or [23 = 15 + (15 − 7)].

Alternatively, half the maintenance dose (312 mg) could be administered more frequently (half the original tau), every 6 h, as calculated using Equation (26).

(26) $$C_{min}=\frac{F\times Dose}{V}\left[\frac{e^{-k_{el}\tau }}{1-e^{-k_{el}\tau }}\right]=\frac{1\times 312}{30}\left[\frac{0.5}{1-0.5}\right]=10.4\;mg/mL$$

Thus, the plasma concentration would fluctuate between about 10.4 to 20 with an average of 15 mg/L.

## 3. Breaking the Law of Superposition

The law of superposition applies when all the disposition processes are linear or first order, this includes absorption, distribution, metabolism, and elimination (ADME) [23,32,34,42,43]. Thus, the concentration after multiple doses can be calculated by adding together the concentrations from each dose. The assumptions underlying the law of superposition are as follows:

1. The dosing is in a range where the pharmacokinetics are linear and dose proportional;
2. The rate and extent of absorption and average clearance are the same for each dosing interval;
3. Each dose acts independently from one another, and the sum of all these dosing events provides the total concentration of the drug in circulation.

**Figure 5:**
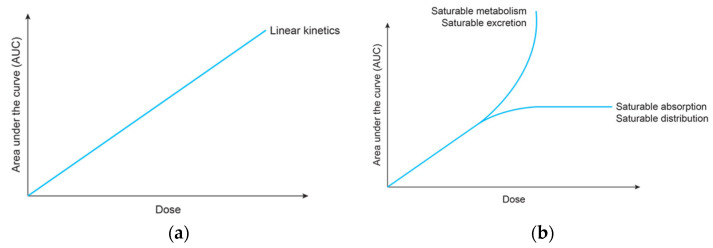
(**a**) Linearity of pharmacokinetics and the validity of the superposition principle, along with (**b**) scenarios where non-linearity in pharmacokinetics leads to the breakdown of this principle.

There are many situations, however, in which the superposition principle does not apply. In these cases, the pharmacokinetics of the drug change after multiple dosing due to various factors, including the changing pathophysiology of the patient, the saturation of the drug carrier system, enzyme induction, and enzyme inhibition [43]. Drugs that follow non-linear pharmacokinetics generally do not have predictable plasma drug concentrations after multiple doses, according to the superposition principle. When the law of superposition is broken, the prediction of the pharmacokinetics, efficacy, and toxicity can become more complicated [23,32,34,42,43]. As described above, the underlying theorem is that the AUC for a single dose will equal the AUC at the steady state. However, the AUC after an oral dose can be affected not only by absorption, but also by protein binding and elimination [43]. Therefore, the AUC at the steady state (AUC_SS_) can either be higher or lower than the AUC after a single dose (AUC_Single Dose_). Figure 5 demonstrates the linearity of pharmacokinetics and the validity of superposition, as well as in instances where non-linearity in pharmacokinetics causes the breakdown of the superposition principle. An overview of possible causes related to various ADME processes, along with examples, is provided in Table 2.

### 3.1. Less than Proportional Increase in AUC (AUC_SS_ < AUC_Single Dose_)

We first consider saturable processes such as absorption, distribution, and elimination. There are at least three processes that can affect drug absorption, namely the saturate–dissolution rate, the GI transit time, and the ability or inability of drugs to cross intestinal barriers [43]. While most drugs are absorbed through passive diffusion, some utilize specific transport processes in the small intestine. When passive diffusion plays a minor role, a less than proportional increase in the area under the curve (AUC) may occur due to the saturation of the active transport process. Drug absorption can be described by a combination of Michaelis–Menten kinetics and first-order kinetics [23,32,34,42,43]. This situation does not always hold. It remains that the absorption of most drugs follows linear kinetics, and the pharmacokinetic parameters describing the absorption of a drug do not change over the therapeutic dose range. However, there are cases where transporters are involved in absorption and, when high doses are utilized (or in overdose situations), the linear range may be exceeded [43]. Sorafenib is an oral tyrosine kinase inhibitor that has been approved for treating advanced renal cell carcinoma and hepatocellular carcinoma. It has been found that the administration of sorafenib at doses exceeding 800 mg/day would result in a less than proportional increase in the area under the curve (AUC) of sorafenib with respect to repeated doses. This phenomenon was attributed to the saturation of the absorption of sorafenib at higher doses. This study has systematically considered and excluded all plausible contributing factors. It has also been demonstrated that partitioning of the daily dose into three discrete administrations would ameliorate the absorption saturation and lead to higher drug exposure at higher drug doses, compared with once or twice daily doses at which the absorption saturation was more notable [57].

Since the concentration of a drug after oral dosing depends on both absorption and elimination, the area under the curve (AUC) represents the combined outcome of these processes. Elimination (clearance) influences this relationship, as a less than proportional increase in AUC at the steady state may result from both increased elimination and decreased absorption. It is generally accepted that only the unbound drug can diffuse across membranes, enabling distribution between the vascular and tissue compartments. Accordingly, changes in drug–protein binding in plasma and tissues can impact the distribution of drugs within the body [32,34,43]. Drug distribution is often measured by the volume of distribution, usually defined as the ratio of the amount of drug in the body to the plasma concentration. The most basic quantitative expression relating to the volume V_d_ of distribution to binding in plasma and tissue is provided by Equation (27).

(27) $$V_{d}=V_{p}+\sum V_{t}\frac{f_{u}}{f_{t}}$$

where V_d_ is the volume of distribution in plasma, V_p_ is the plasma volume, V_t_ is the tissue volume, and f_u_ and f_t_ are the fraction of the unbound drug in the plasma and tissue, respectively.

It is clear that the volume of distribution depends on plasma protein binding, but also on tissue binding. For drugs with low clearance, protein binding significantly influences drug elimination, and total body clearance is directly proportional to the unbound fraction in plasma [23,34,39,43]. A drug can exhibit concentration-dependent protein binding, with the unbound fraction changing [43]. A less than proportional increase in the AUC due to the saturation of protein-binding sites in plasma may result in an increase in the free fraction, which will result in enhanced clearance. The AUC_SS_ would be increased less than proportionally, and the bioavailability reduced; thus, the AUC_SS_ would be less than the AUC_Single Dose_. Assuming a drug binds to n discrete sites on a protein molecule, with all sites having the same affinity for the drug and acting independently, the fraction of the unbound drug, fu, can be described by Equation (28).

(28) $$f_{u}=\frac{K_{d}+D_{f}}{{np}_{t}+K_{d}+D_{f}}$$

where K_d_ is the dissociation constant at the equilibrium, D_f_ and p_t_ are the concentration of the unbound drug and total protein (bound and unbound), respectively, and n is the number of binding sites on the protein molecule. The binding of a drug to a protein is determined by four factors: the drug concentration, the protein concentration, the number of binding sites per protein molecule, and the dissociation constant [43]. Irrespective of the affinity, the maximum possible binding capacity is the product of the molar concentration of the protein and the number of binding sites per protein molecule. Mathematically, it can be observed that with multiple dosing, an elevated unbound fraction in plasma, fu, becomes apparent in plasma. This results in an increased volume of distribution, lowered plasma concentrations, and a less than proportional increase in the AUC. Vismodegib (Erivedge™, GDC-0449; Genentech, South San Francisco, CA, USA) is an approved drug in the United States for the treatment of advanced basal cell carcinoma (BCC). An evaluation of the intravenous pharmacokinetic (PK) parameters, following both single and multiple oral dosing regimens, showed increased clearance and an increased volume of distribution, with values 81% and 63% higher, respectively, following repeated dosing. Moreover, a significant reduction in bioavailability (77% lower) was observed with multiple doses. Intriguingly, continuous daily dosing of vismodegib led to a 2.4-fold increase in the unbound fraction compared to a single dose [58].

In tissues, drugs can bind to non-specific binding proteins, enzymes, and receptors [43]. Enzymes and receptors are particularly important pharmacologically, as they contribute to the therapeutic effect. However, distinguishing these from non-specific binding sites is challenging because the binding capacity of enzymes and receptors are often low and overshadowed by non-specific binding. Similar to plasma protein binding, tissue protein binding can become saturated as drug concentrations increase over time, resulting in changes in the volume of distribution. However, the saturation of tissue binding and of plasma protein binding can occur simultaneously [43]. The volume of distribution is directly related to plasma protein binding and inversely related to tissue binding, so changes in one type of binding can counterbalance the other. This complexity is further compounded by the fact that different tissues may not become saturated to the same extent. Consequently, the volume of distribution of a drug may not be significantly affected by changes in tissue binding, depending on the degree of non-linear tissue binding. Thus, it is essential to evaluate the non-linearity of tissue binding by examining the ratio of the drug concentration in a given tissue to that in plasma. At equilibrium, the tissue/plasma concentration ratio should match the ratio of the unbound fraction in plasma to the unbound fraction in a non-eliminating tissue [43]. In eliminating tissues, it is more complex; the ratio may be influenced by blood flow to the tissue and the eliminating process. Moreover, elimination could also be impacted by changes in intrinsic clearance (CL_int’_). Enzyme autoinduction is a time-dependent phenomenon, where the elimination of a drug increases with multiple doses [43]. Thus, an AUC_SS_ less than the AUC_Single Dose_ may be observed. One such example is the anticonvulsant carbamazepine (CBZ), that is known to auto-induce its own metabolism. Throughout the course of multiple 200 mg doses of CBZ, the daily trough CBZ levels consistently declined, reaching their lowest point after two weeks. Notably, the observed CBZ concentration was only 53.9% of what had been predicted under the assumption of no autoinduction. Furthermore, the total CBZ clearance following multiple doses was higher than after a single dose. Digging deeper into CBZ pharmacokinetics, considerable increases in the AUCs of CBZ metabolites during multiple-dose administration were noted. These changes were accompanied by increased urinary excretion of these metabolites compared to CBZ, all due to the phenomenon of autoinduction [59].

Phenytoin serves as another illustrative case, akin to carbamazepine. Like carbamazepine, phenytoin, an antiepileptic medication, also exhibits violation of the law of superposition due to enzyme autoinduction. In the case of phenytoin, after the third dose, the calculated AUC was found to be statistically significantly smaller than that observed after the initial dose. This phenomenon was attributed to the ability of phenytoin to induce its own metabolism. Consequently, this autoinduction led to an increased rate of elimination, which in turn resulted in a reduction in the AUC following multiple doses [60].

### 3.2. More than Proportional Increase in AUC (AUC_SS_ > AUC_Single Dose_)

A pharmacokinetic situation in which an increase in the AUC at the steady state is greater than after a single dose is also possible. In this case, a lack of proportionality is more likely due to a decrease in elimination than to an increase in absorption [43]. However, in certain instances, absorption can increase with higher doses, due to specific mechanisms.

The more than proportional increase in the AUC is likely due to non-linear clearance. This disproportionate increase in the AUC is most likely attributable to decreased elimination clearance after multiple doses [32,43]. For drugs with a high level of extraction in the liver, a significant first-pass effect occurs. If this first-pass effect diminishes with multiple dosing, it can lead to a disproportionate increase in the AUC. This increase is due to both a decrease in elimination and an increase in bioavailability, the latter resulting from the saturation of hepatic metabolism during the initial passage of the drug through the liver [43]. The disproportionate increase in the AUC with multiple oral doses can be linked to enhanced bioavailability resulting from a reduced first-pass effect. The effects of capacity-limited metabolism are more apparent under steady-state conditions than after a single dose. This is exemplified by the relationship between the plasma phenytoin concentration at the steady state and the administered dose. At the steady state, the plasma concentration of phenytoin increases more than proportionally in terms of the rate of the input [29,44]. Verapamil, a drug used to treat cardiovascular conditions, exhibits low oral bioavailability, primarily due to extensive pre-systemic (first pass) liver elimination. When the pharmacokinetics of verapamil were assessed, it was observed that the AUC at the steady state (1999 ± 435 ng/mL h) exceeded that recorded after the initial dose (788 ± 224 ng/mL h). That increase in AUC was attributed to the saturation of the first-pass effect that occurred with multiple dosing. As the first-pass effect became saturated, a greater proportion of the drug managed to evade pre-systemic elimination. That, in turn, led to the accumulation of verapamil and an increase in the AUC with multiple doses [61,62].

Drugs are eliminated from the body through various processes, including biotransformation and excretion. The efficiency with which the body eliminates a drug is commonly expressed as the total clearance, which is the sum of the individual clearances of different organs, occurring simultaneously. CL_int_ is the intrinsic clearance, a measure of the intracellular removal of a drug, described by the Michaelis–Menten equation, where V is the capacity of the enzyme or carrier systems, K is the Michaelis constant, and *C* is the unbound drug concentration [39]. It is evident that the CL_int_ remains constant when the concentration is lower than K, with a limited value of V/K. However, non-linearity arises when the drug concentration approaches the value of K. Since drug elimination typically involves multiple processes, determining whether elimination is linear or non-linear becomes increasingly complex as the number of these processes increases [43]. Depending on the relative contribution of saturable pathways to overall elimination, non-linearity may not be easily discernible.

When saturation occurs, the accumulation of the drug will be greater at the steady state than predicted from a single dose [21,42,43]. Drug elimination often exhibits zero-order kinetics at high concentrations and first-order kinetics at low concentrations, a phenomenon known as concentration-dependent kinetics. At multiple doses, which lead to a higher plasma concentration, zero-order kinetics are observed. Conversely, at lower doses, the kinetics are linear, or first order. This pattern is particularly common with drugs that are extensively metabolized [43]. A typical characteristic of enzymatic reactions and active transport is their limited capacity. The liver has a finite amount of enzymes, establishing a maximum rate at which metabolism can occur. Additionally, the rate of metabolism can be further constrained by the limited availability of co-substances or co-factors necessary for the enzymatic process [43].

Most of our understanding of enzyme kinetics comes from in vitro studies, where the concentrations of the substrates, enzymes, and co-factors are meticulously controlled. Numerous factors are involved in in vivo studies, making it difficult to isolate each one and assess it in detail. Nevertheless, the fundamental principles of enzyme kinetics are applicable to pharmacokinetics. At single dose concentrations, where K_m_ > C_p_, K_m_ + C_p_ is approximately equal to K_m_, as described in Equation (29).

(29) $$\frac{\partial C_{p}}{\partial t}=\frac{V_{m}}{K_{m}}C_{p}$$

where V_m_/K_m_ is a first-order elimination rate constant and the whole equation now looks like that for first-order elimination.

Therefore, at a low plasma concentration following a single dose, first-order kinetics would be expected. This is a typical situation for most drugs, as the K_m_ is usually larger than the plasma concentration achieved. However, a high concentration at multiple dosing is achieved, that is C_p_ > K_m_, then K_m_ + C_p_ is approximately equal to C_p_ leading to a zero-order elimination process, as described in Equation (30).

(30) $$\frac{\partial C_{p}}{\partial t}=\frac{V_{m}\times C_{p}}{C_{p}}=-V_{m}$$

At high steady-state plasma concentrations, zero-order or concentration-independent kinetics occur. The presence of saturation kinetics can be significant when multiple doses of certain drugs are given, or in cases of overdose. At the steady state, the effective elimination rate constant is reduced, leading to excessive drug accumulation if the saturation kinetics are not properly understood.

The renal excretion of drugs usually involves three processes: glomerular filtration, renal tubular secretion, and reabsorption from the renal tubular lumen [43]. Glomerular filtration is a passive process that depends on the unbound concentration of a drug in plasma. The relationship between renal clearance CL_R_ and these processes is described in Equation (31).

(31) $${\mathrm{CL}}_{R}=f_{u}\times GFR+CL$$

where GFR, CL, and CL_R_ are the glomerular filtration rate, secretion clearance, and reabsorption clearance, respectively, and f_u_ is the unbound fraction of the drug in plasma. When the CL_R_ and GFR of a drug are much greater than one, it indicates that renal tubular secretion of the drug is occurring [43]. Conversely, when the ratio is much less than one, it suggests reabsorption of the drug from the tubular lumen. Renal tubular secretion is a specialized and saturable process, whereas tubular reabsorption can occur through passive diffusion or active transport, while active reabsorption is also saturable [43]. In addition, there are processes whose rates are affected by pharmacological actions of the drug itself. Elimination could also be affected through changes in intrinsic clearance (CL_int’_). Enzyme inhibition can be a time-dependent phenomenon, in which the elimination clearance of a drug decreases following multiple doses and the decrease in clearance is significant compared to a single dose [43]. Thus, the AUC_SS_ would be higher than the AUC_Single Dose_. Propranolol and itraconazole provide examples of how changes in intrinsic clearance (CL_int’_) can affect elimination. When administered in multiple doses, these drugs have been found to inhibit their own metabolizing enzymes. That inhibition resulted in a decrease in their hepatic clearance and led to an increase in the AUC at the steady state [63,64].

### 3.3. Other Factors Affecting Adherence to the Law of Superposition

The reasons discussed in the previous two sections pertain to situations where the law of superposition may not accurately characterize drug behavior due to drug-related factors. However, in other instances, a variety of factors can play a role. Patient-related causes that can lead to deviations from the law of superposition include non-compliance, irregular dosing schedules, and inconsistent dosing amounts [19,20,65]. If the patient does not take the prescribed dosing amount or does not adhere to the dosing schedule, the drug concentration–time curve can exhibit an increase in fluctuation. Similarly, variations in the dosage amount taken by the patient may result in varying drug levels in the body, which can impact the pharmacokinetic behavior of some drugs. Inconsistent dosing sizes may result in varying drug levels in the body, which can lead to the appearance of the superposition principle not being upheld. In all the described cases, the variability in drug administration may cause deviations from the expected linear pharmacokinetic behavior described by the law of superposition.

When patients take medications, some drugs may interact with specific components in the food they ingest or those released during the digestive process, leading to a reduction in the anticipated absorption [66,67]. When patients deviate from prescribed dosage instructions, these interactions can impact the expected pharmacokinetic behavior of drugs, especially when multiple doses are involved. The impact of meals on drug absorption is multifaceted. In particular, the concurrent administration of certain drugs with food has been shown to enhance their absorption. This phenomenon is particularly relevant for drugs that rely on surfactants to facilitate their absorption [66]. Also, lipophilic drugs when taken with a fatty meal can follow another absorption pathway, bypassing the traditional portal vein route and instead entering the systemic circulation via the lymphatic system, alongside absorbed dietary fats [68,69].

Drug–drug interactions stand as another influential factor that can disrupt the adherence to the law of superposition for certain drugs. When certain drugs are co-administered with others, several intricate interactions can come into play, ultimately impacting their pharmacokinetic behavior. One common scenario involves competitive interactions, where co-administered drugs may compete for the same transporters responsible for drug absorption or elimination [70]. This competitive struggle for transporters can lead to deviations from the expected linear pharmacokinetics of the involved drugs. Furthermore, drug interactions can also affect metabolizing enzymes [71]. Some drugs may induce or inhibit these enzymes, which play critical roles in processes like pre-systemic first-pass metabolism and overall liver clearance [72,73]. These enzyme-related changes can significantly influence the pharmacokinetic profile of drugs, causing it to deviate from the anticipated linear behavior described by the law of superposition. Another intricate effect arises from the interaction between co-administered drugs with high plasma protein-binding affinity [74,75]. When two drugs that both extensively bind to plasma proteins are taken together, they can engage in what is known as protein-binding substitution. This process can influence the distribution and clearance of both drugs, as they essentially compete for available protein-binding sites.

## 4. Conclusions

The pharmacokinetics and area under the concentration–time curve of the majority of drugs after administration of a single dose can be described by first-order or linear processes and can be used to predict a similar steady-state area under the concentration–time curve exposure and, thus, follow the law of superposition. However, there are a number of scenarios and situations where drugs could display behaviors after multiple dosing that leads to capacity-limited or saturation non-linear kinetics and the law of superposition is overruled and not followed. As a consequence, when ADME processes are saturated, the AUC at the steady state may result in substantial changes to the plasma concentration, possibly leading to changes in the pharmacodynamics effect or a toxic effect. Thus, a sound understanding of the principles and influence of dosing (single or multiple dosing) on pharmacokinetics is important in the analysis of pharmacokinetic data by analysts during drug development.

## Figures and Tables

**Table 1:** Concentration of Canadamycin (a hypothetical drug) in mg/L before each subsequent multiple dose is administered.

Dose	Canadamycin (mg/L)	Start Multiple	Trough End (r)	Time (h)	Concentration Lost during Dosage Interval
1	20.00	20.00	5.0	8	15.00
2	5.00	25.00	6.25	16	18.75
3	1.25	26.25	6.56	24	19.69
4	0.3125	26.56	6.64	32	19.92
5	0.078125	26.64	6.66	40	19.98
6	0.01953125	26.66	6.67	48	19.99
7	0.004882812	26.67	6.67	54	20.00

**Table 2:** Primary potential causes for deviations from the law of superposition in various ADME processes, with examples.

ADME Process		Main Related Causes of Non-Linearity		Examples
Absorption		Carrier-mediated transport		Gabapentin [46], Metformin [47]
		Saturable pre-systemic loss	Gut metabolism	Verapamil [48]
			First-pass metabolism	Propranolol [49]
Distribution		Saturable plasma protein binding		Paclitaxel [50]
		Saturable tissue binding		Imipramine [51]
Elimination	Metabolism	Saturable metabolism		Theophylline [52]
		Enzyme induction		Carbamazepine [53]
	Excretion	Saturable renal excretion	Saturable active secretion	Zidovudine [54]
			Saturable reabsorption	Methotrexate [55]
		Saturable biliary excretion		Tetracycline [56]

## Data Availability

Not applicable.

## References

[1] Garza I., Swanson J.W. (2006). Prophylaxis of migraine. Neuropsychiatr. Dis. Treat..

[2] Ong J.J.Y., De Felice M. (2018). Migraine Treatment: Current Acute Medications and Their Potential Mechanisms of Action. Neurotherapeutics.

[3] Diener H.-C., Holle-Lee D., Nägel S., Dresler T., Gaul C., Göbel H., Heinze-Kuhn K., Jürgens T., Kropp P., Meyer B. (2019). Treatment of migraine attacks and prevention of migraine: Guidelines by the German Migraine and Headache Society and the German Society of Neurology. Clin. Transl. Neurosci..

[4] Do T.P., Guo S., Ashina M. (2019). Therapeutic novelties in migraine: New drugs, new hope?. J. Headache Pain.

[5] Borbély A.A., Mattmann P., Loepfe M., Fellmann I., Gerne M., Strauch I., Lehmann D. (1983). A single dose of benzodiazepine hypnotics alters the sleep EEG in the subsequent drug-free night. Eur. J. Pharmacol..

[6] Johnson L.C., Chernik D.A. (1982). Sedative-hypnotics and human performance. Psychopharmacology.

[7] Malpas A., Rowan A.J., Boyce C.R., Scott D.F. (1970). Persistent behavioural and electroencephalographic changes after single doses of nitrazepam and amylobarbitone sodium. Br. Med. J..

[8] Kornetsky C., Vates T.S., Kessler E.K. (1959). A comparison of hypnotic and residual psychological effects of single doses of chlorpromazine and secobarbital in man. J. Pharmacol. Exp. Ther..

[9] Eckburg P.B., Jain A., Walpole S., Moore G., Utley L., Manyak E., Dane A., Melnick D. (2019). Safety, Pharmacokinetics, and Food Effect of Tebipenem Pivoxil Hydrobromide after Single and Multiple Ascending Oral Doses in Healthy Adult Subjects. Antimicrob. Agents Chemother..

[10] Chua H.C., Tse A., Smith N.M., Mergenhagen K.A., Cha R., Tsuji B.T. (2021). Combatting the Rising Tide of Antimicrobial Resistance: Pharmacokinetic/Pharmacodynamic Dosing Strategies for Maximal Precision. Int. J. Antimicrob. Agents.

[11] Xie J., Roberts J.A., Lipman J., Cai Y., Wang H., Zhao N., Xu X., Yang S., Li Y., Zhang K. (2020). Pharmacokinetic/pharmacodynamic adequacy of polymyxin B against extensively drug-resistant Gram-negative bacteria in critically ill, general ward and cystic fibrosis patient populations. Int. J. Antimicrob. Agents.

[12] Bunke C.M., Aronoff G.R., Luft F.C. (1983). Pharmacokinetics of common antibiotics used in continuous ambulatory peritoneal dialysis. Am. J. Kidney Dis..

[13] Kang W.Y., Lee H.W., Gwon M.R., Cho S., Shim W.S., Lee K.T., Yang D.H., Seong S.J., Yoon Y.R. (2020). A Pharmacokinetic Drug Interaction Between Fimasartan and Linagliptin in Healthy Volunteers. Drug Des. Dev. Ther..

[14] Moon S.J., Yu K.S., Kim M.G. (2020). An Assessment of Pharmacokinetic Interaction Between Lobeglitazone and Sitagliptin After Multiple Oral Administrations in Healthy Men. Clin. Ther..

[15] Ghim J.L., Phuong N.T.T., Kim M.J., Kim E.J., Song G.S., Ahn S., Shin J.G., Kim E.Y. (2019). Pharmacokinetics of fixed-dose combination of atorvastatin and metformin compared with individual tablets. Drug Des. Dev. Ther..

[16] Sheng L., Cao W., Lin P., Chen W., Xu H., Zhong C., Yuan F., Chen H., Li H., Liu C. (2021). Safety, Tolerability and Pharmacokinetics of Single and Multiple Ascending Doses of Benfotiamine in Healthy Subjects. Drug Des. Dev. Ther..

[17] Kim J.R., Kim S., Huh W., Ko J.W. (2018). No pharmacokinetic interactions between candesartan and amlodipine following multiple oral administrations in healthy subjects. Drug Des. Dev. Ther..

[18] Vaidyanathan S., Jarugula V., Dieterich H.A., Howard D., Dole W.P. (2008). Clinical pharmacokinetics and pharmacodynamics of aliskiren. Clin. Pharmacokinet..

[19] Wang W., Husan F., Chow S.C. (1996). The impact of patient compliance on drug concentration profile in multiple doses. Stat. Med..

[20] Wang W., Millard S.P., Krause A. (2001). Patient Compliance and its Impact on Steady State Pharmacokinetics. Applied Statistics in the Pharmaceutical Industry: with Case Studies Using S-Plus.

[21] van Rossum J.M. (1968). Pharmacokinetics of accumulation. J. Pharm. Sci..

[22] Wang W., Ouyang S.P. (1998). The formulation of the principle of superposition in the presence of non-compliance and its applications in multiple dose pharmacokinetics. J. Pharmacokinet. Biopharm..

[23] Lin J.H. (1994). Dose-dependent pharmacokinetics: Experimental observations and theoretical considerations. Biopharm. Drug Dispos..

[24] Cutler D.J. (1978). Numerical deconvolution by least squares: Use of prescribed input functions. J. Pharmacokinet. Biopharm..

[25] Cutler D.J. (1978). Numerical deconvolution by least squares: Use of polynomials to represent the input function. J. Pharmacokinet. Biopharm..

[26] Fernández-Campos F., Ferrero C., Colom H., Jiménez-Castellanos M.R. (2015). In vivo absorption behaviour of theophylline from starch-methyl methacrylate matrix tablets in beagle dogs. Int. J. Pharm..

[27] Chiou W.L. (1979). Rapid compartment- and model-independent estimation of times required to attain various fractions of steady-state plasma level during multiple dosing of drugs obeying superposition principle and having various absorption or infusion kinetics. J. Pharm. Sci..

[28] Gupta P., Hutmacher M.M., Frame B., Miller R. (2008). An alternative method for population pharmacokinetic data analysis under noncompliance. J. Pharmacokinet. Pharmacodyn..

[29] Ma L. (2013). Analysis of Nonlinear Pharmacokinetic Systems and the Nonlinear Disposition of Phenylbutazone in Equine (Horses). Ph.D. Thesis.

[30] McCoy A.T., Bartels M.J., Rick D.L., Saghir S.A. (2012). TK Modeler version 1.0, a Microsoft^®^ Excel^®^-based modeling software for the prediction of diurnal blood/plasma concentration for toxicokinetic use. Regul. Toxicol. Pharmacol..

[31] Mengozzi G., Intorre L., Bertini S., Giorgi M., Soldani G. (1998). Comparative bioavailability of two sustained-release theophylline formulations in the dog. Pharmacol. Res..

[32] Thron C.D. (1974). Linearity and superposition in pharmacokinetics. Pharmacol. Rev..

[33] Tuntland T., Ethell B., Kosaka T., Blasco F., Zang R.X., Jain M., Gould T., Hoffmaster K. (2014). Implementation of pharmacokinetic and pharmacodynamic strategies in early research phases of drug discovery and development at Novartis Institute of Biomedical Research. Front. Pharmacol..

[34] van Rossum J.M., de Bie J.E. (1989). Systems dynamics in clinical pharmacokinetics. An introduction. Clin. Pharmacokinet..

[35] Peletier L.A., de Winter W. (2017). Impact of saturable distribution in compartmental PK models: Dynamics and practical use. J. Pharmacokinet. Pharmacodyn..

[36] Gabrielsson J., Meibohm B., Weiner D. (2016). Pattern Recognition in Pharmacokinetic Data Analysis. AAPS J..

[37] Mehrotra N., Gupta M., Kovar A., Meibohm B. (2007). The role of pharmacokinetics and pharmacodynamics in phosphodiesterase-5 inhibitor therapy. Int. J. Impot. Res..

[38] Shen J., Boeckmann A., Vick A. (2012). Implementation of dose superimposition to introduce multiple doses for a mathematical absorption model (transit compartment model). J. Pharmacokinet. Pharmacodyn..

[39] Siwale R.C., Sani S.N., Ducharme M.P., Shargel L. (2022). Multiple-Dosage Regimens. Applied Biopharmaceutics & Pharmacokinetics.

[40] Mehvar R. (2008). Dependence of time to reach steady-state on the length of dosage interval. Ann. Pharmacother..

[41] Hieb B.R., Shrewsbury B. (1980). Consecutive intravenous infusions: Simulation of two compartment pharmacokinetic drugs. Comput. Programs Biomed..

[42] Brocks D.R., Mehvar R. (2010). Rate and extent of drug accumulation after multiple dosing revisited. Clin. Pharmacokinet..

[43] van Rossum J.M., van Lingen G., Burgers J.P.T. (1983). Dose-dependent pharmacokinetics. Pharmacol. Ther..

[44] Nation R.L., Evans A.M., Milne R.W. (1990). Pharmacokinetic drug interactions with phenytoin (Part I). Clin. Pharmacokinet..

[45] Yáñez J.A., Remsberg C.M., Sayre C.L., Forrest M.L., Davies N.M. (2011). Flip-flop pharmacokinetics–delivering a reversal of disposition: Challenges and opportunities during drug development. Ther. Deliv..

[46] Stewart B.H., Kugler A.R., Thompson P.R., Bockbrader H.N. (1993). A saturable transport mechanism in the intestinal absorption of gabapentin is the underlying cause of the lack of proportionality between increasing dose and drug levels in plasma. Pharm. Res..

[47] Proctor W.R., Bourdet D.L., Thakker D.R. (2008). Mechanisms underlying saturable intestinal absorption of metformin. Drug Metab. Dispos..

[48] Hanada K., Ikemi Y., Kukita K., Mihara K., Ogata H. (2008). Stereoselective first-pass metabolism of verapamil in the small intestine and liver in rats. Drug Metab. Dispos..

[49] Kalam M.N., Rasool M.F., Rehman A.U., Ahmed N. (2020). Clinical pharmacokinetics of propranolol hydrochloride: A review. Curr. Drug Metab..

[50] Karlsson M.O., Molnar V., Freijs A., Nygren P., Bergh J., Larsson R. (1999). Pharmacokinetic models for the saturable distribution of paclitaxel. Drug Metab. Dispos..

[51] Eling T.E., Pickett R.D., Orton T.C., Anderson M.W. (1975). A study of the dynamics of imipramine accumulation in the isolated perfused rabbit lung. Drug Metab. Dispos..

[52] Ha H.R., Chen J., Freiburghaus A.U., Follath F. (1995). Metabolism of theophylline by cDNA-expressed human cytochromes P-450. Br. J. Clin. Pharmacol..

[53] Bernus I., Dickinson R.G., Hooper W.D., Eadie M.J. (1996). Dose-dependent metabolism of carbamazepine in humans. Epilepsy Res..

[54] Patel B.A., Chu C.K., Boudinot F.D. (1989). Pharmacokinetics and saturable renal tubular secretion of zidovudine in rats. J. Pharm. Sci..

[55] Hendel J., Nyfors A. (1984). Nonlinear renal elimination kinetics of methotrexate due to saturation of renal tubular reabsorption. Eur. J. Clin. Pharmacol..

[56] Babu E., Takeda M., Narikawa S., Kobayashi Y., Yamamoto T., Cha S.H., Sekine T., Sakthisekaran D., Endou H. (2002). Human organic anion transporters mediate the transport of tetracycline. Jpn. J. Pharmacol..

[57] Hornecker M., Blanchet B., Billemont B., Sassi H., Ropert S., Taieb F., Mir O., Abbas H., Harcouet L., Coriat R. (2012). Saturable absorption of sorafenib in patients with solid tumors: A population model. Investig. New Drug..

[58] Graham R.A., Hop C.E.C.A., Borin M.T., Lum B.L., Colburn D., Chang I., Shin Y.G., Malhi V., Low J.A., Dresser M.J. (2012). Single and multiple dose intravenous and oral pharmacokinetics of the hedgehog pathway inhibitor vismodegib in healthy female subjects. Br. J. Clin. Pharmacol..

[59] Yoon Y.-R., Shin J.-G., Cha I.-J., Kim K.-A., Shim J.-C., Kim Y.-H., Shin J.-B. (1996). Pharmacokinetic analysis on autoinduction of carbamazepine metabolism. J. Korean Soc. Clin. Pharmacol. Ther..

[60] Chetty M., Miller R., Seymour M.A. (1998). Phenytoin auto-induction. Ther. Drug Monit..

[61] Shand D.G., Hammill S.C., Aanonsen L., Pritchett E.L.C. (1981). Reduced verapamil clearance during long-term oral administration. Clin. Pharmacol. Ther..

[62] Abernethy D.R., Wainer I.W., Anacleto A.I. (2000). Verapamil metabolite exposure in older and younger men during steady-state oral verapamil administration. Drug Met. Dispos..

[63] Lalonde R.L., Pieper J.A., Straka R.J., Bottorff M.B., Mirvis D.M. (1987). Propranolol pharmacokinetics and pharmacodynamics after single doses and at steady-state. Eur. J. Clin. Pharmacol..

[64] Barone J.A., Koh J.G., Bierman R.H., Colaizzi J.L., Swanson K.A., Gaffar M.C., Moskovitz B.L., Mechlinski W., Van de Velde V. (1993). Food interaction and steady-state pharmacokinetics of itraconazole capsules in healthy male volunteers. Antimicrob. Agents Chemother..

[65] Vrijens B., Goetghebeur E. (1999). The impact of compliance in pharmacokinetic studies. Stat. Methods Med. Res..

[66] Martinez M.N., Amidon G.L. (2002). A mechanistic approach to understanding the factors affecting drug absorption: A review of fundamentals. J. Clin. Pharmacol..

[67] Singh B.N. (1999). Effects of food on clinical pharmacokinetics. Clin. Pharmacokinet..

[68] Gershkovich P., Hoffman A. (2007). Effect of a high-fat meal on absorption and disposition of lipophilic compounds: The importance of degree of association with triglyceride-rich lipoproteins. Eur. J. Pharm. Sci..

[69] Yousef M., Silva D., Bou-Chacra N., Davies N.M., Löbenberg R. (2021). The lymphatic system: A sometimes forgotten compartment in pharmaceutical sciences. J. Pharm. Pharm. Sci..

[70] König J., Müller F., Fromm M.F. (2013). Transporters and drug-drug interactions: Important determinants of drug disposition and effects. Pharmacol. Rev..

[71] Tornio A., Filppula A.M., Niemi M., Backman J.T. (2019). Clinical studies on drug–drug interactions involving metabolism and transport: Methodology, pitfalls, and interpretation. Clin. Pharmacol. Ther..

[72] Hall S.D., Thummel K.E., Watkins P.B., Lown K.S., Benet L.Z., Paine M.F., Mayo R.R., Turgeon D.K., Bailey D.G., Fontana R.J. (1999). Molecular and physical mechanisms of first-pass extraction. Drug Met. Dispos..

[73] Bachmann K. (2009). Drug–drug interactions with an emphasis on drug metabolism and transport. Pharmacology Principles and Practice, Hacker, M., Messer, W., Bachmann, K., Eds..

[74] DeVane C.L. (2002). Clinical significance of drug binding, protein binding, and binding displacement drug interactions. Psychopharmacol. Bull..

[75] Heuberger J., Schmidt S., Derendorf H. (2013). When is protein binding important?. J. Pharm. Sci..

