# Correction: Yousef et al. Upholding or Breaking the Law of Superposition in Pharmacokinetics. *Biomedicines* 2024, *12*, 1843

**DOI:** 10.3390/biomedicines13081797

**Published:** 2025-07-23

**Authors:** Malaz Yousef, Jaime A. Yáñez, Raimar Löbenberg, Neal M. Davies

**Affiliations:** 1 Faculty of Pharmacy and Pharmaceutical Sciences, University of Alberta, Edmonton, AB T6G 2E1, Canada; malaz@ualberta.ca (M.Y.); raimar@ualberta.ca (R.L.); 2 Escuela de Posgrado, Universidad Internacional Iberoamericana, Campeche 24560, Mexico; jaime.yanez@unini.edu.mx

## Error in Table

In the original publication [1], there was a mistake in Table 1 as published. In Table 1, the row labelled “Dose 1” appears twice; once at the top and once again at the bottom (after Dose 7). This repeated entry was unintentional and should be removed. The correct table should end at Dose 7, and the repeated Dose 1 row at the bottom is redundant and may cause confusion. The corrected Table 1 appears below. The authors state that the scientific conclusions are unaffected. This correction was approved by the Academic Editor. The original publication has also been updated.

## Text Correction

There was an error in the original publication [1]. There is an inconsistency between the data presented in Table 1 and the corresponding text in Section 2.2.

A correction has been made to Section 2, specifically in Section 2.2, in the first paragraph:

“For example, administering a third dose at the 16 h mark will also produce an initial concentration of 20 mg/L. The concentration remaining from the second dose would be 5 mg/L, and the concentration remaining from the first dose would be 1.25 mg/L. When these concentrations are combined, the observed total concentration will be approximately 26.25 mg/L, calculated as 20 mg/L (from the third dose) + 5 mg/L (remaining from the second dose) + 1.25 mg/L (remaining from the first dose). This additive process of combining concentrations from multiple doses to determine the observed concentration exemplifies the principle of superposition [21,42].”

The authors state that the scientific conclusions are unaffected. This correction was approved by the Academic Editor. The original publication has also been updated.

## Figures and Tables

**Table 1:** Concentration of Canadamycin (a hypothetical drug) in mg/L before each subsequent multiple dose is administered.

Dose	Canadamycin (mg/L)	Start Multiple	Trough End (r)	Time (h)	Concentration Lost during Dosage Interval
1	20.00	20.00	5.0	8	15.00
2	5.00	25.00	6.25	16	18.75
3	1.25	26.25	6.56	24	19.69
4	0.3125	26.56	6.64	32	19.92
5	0.078125	26.64	6.66	40	19.98
6	0.01953125	26.66	6.67	48	19.99
7	0.004882812	26.67	6.67	54	20.00

## References

[1] Yousef M., Yáñez J.A., Löbenberg R., Davies N.M. (2024). Upholding or Breaking the Law of Superposition in Pharmacokinetics. Biomedicines.

